# Description of *Pseudomonas imrae* sp. nov., carrying a novel class C β-lactamase gene variant, isolated from gut samples of Atlantic mackerel (*Scomber scombrus*)

**DOI:** 10.3389/fmicb.2025.1530878

**Published:** 2025-04-14

**Authors:** Francisco Salvà-Serra, Priyank Nimje, Beatriz Piñeiro-Iglesias, Leonarda Achá Alarcón, Sofia Cardew, Elisabeth Inganäs, Susanne Jensie-Markopoulos, Maria Ohlén, Hanna-Sophia Sailer, Christel Unosson, Víctor Fernández-Juárez, Cesar O. Pacherres, Michael Kühl, Edward R. B. Moore, Nachiket P. Marathe

**Affiliations:** ^1^Department of Infectious Diseases, Institute for Biomedicine, Sahlgrenska Academy, University of Gothenburg, Gothenburg, Sweden; ^2^Department of Clinical Microbiology, Sahlgrenska University Hospital, Gothenburg, Sweden; ^3^Culture Collection University of Gothenburg (CCUG), Department of Clinical Microbiology, Sahlgrenska University Hospital and Sahlgrenska Academy, University of Gothenburg, Gothenburg, Sweden; ^4^Centre for Antibiotic Resistance Research (CARe), University of Gothenburg, Gothenburg, Sweden; ^5^Methodology Textiles and Medical Technology, Division Materials and Production, RISE Research Institutes of Sweden, Gothenburg, Sweden; ^6^Institute of Marine Research (IMR), Bergen, Norway; ^7^Department of Biology and Nordic Center for Earth Evolution (NordCEE), University of Southern Denmark, Odense, Denmark; ^8^Marine Biological Section, Department of Biology, University of Copenhagen, Copenhagen, Denmark

**Keywords:** *Pseudomonas fluorescens* group, *Pseudomonas gessardii* subgroup, β-lactamase novel variant, novel species, polyphasic taxonomy, phylogenomics, whole-genome sequencing

## Abstract

Three β-lactam resistant bacterial strains isolated from gut samples of wild Atlantic mackerel (*Scomber scombrus*) collected from the northern North Sea were characterized by polyphasic analyses. The strains were determined to belong to the genus *Pseudomonas* but could not be assigned to a known species. The nearly-complete 16S rRNA gene sequence showed the highest similarity (99.9%) to four different species, although partial *rpoD* sequence exhibited relatively low similarities to *Pseudomonas proteolytica* (93.4%) and other *Pseudomonas* spp. Genome sequencing and subsequent digital DNA–DNA hybridization (dDDH), average nucleotide identity (ANI) analysis and core genome analysis confirmed that these strains represent a novel species within the genus *Pseudomonas*. The three strains demonstrated ANIb values >99.5% with each other, confirming that all three strains (CCUG 74779^T^ = CECT 30571^T^, CCUG 74780 and CCUG 74781) belong to the same genomospecies. Phylogenomic analysis confirmed that the strains form a distinct genomic clade, representing a novel taxonomic species, for which the name *Pseudomonas imrae* sp. nov., is proposed, with strain CCUG 74779^T^ (=CECT 30571^T^) designated as the type strain. We report the complete genome sequence of the type strain of *P. imrae* sp. nov. and show that it carries a gene encoding a novel variant of a chromosomally-encoded class C β-lactamase, which has been designated as PFL-7.

## Introduction

The species of the genus *Pseudomonas* are aerobic, oxidase-positive, Gram-negative bacilli. They are present in a wide variety of environments, including soil, water, plants, humans, and other animals, and have a broad metabolic diversity (Palleroni, 2015). Many *Pseudomonas* species exhibit beneficial functions in the environment, including biodegradation of complex compounds and enhanced plant growth (Dong et al., 2023; Pieterse et al., 2021; Peix et al., 2018), while some species are opportunistic pathogens, with *P. aeruginosa* being the most virulent and clinically-relevant human pathogen of the genus (Qin et al., 2022; Ikuta et al., 2022). The genus *Pseudomonas* is highly diverse and taxonomically complex, comprising more than 340 validly published species names,^1^ which can be further subdivided into several groups and subgroups (Lalucat et al., 2020; Lalucat et al., 2022). Some members of the genus *Pseudomonas* have been recently reclassified into several novel genera, such as *Atopomonas*, *Halopseudomonas, Stutzerimonas* and *Trinickia*, by whole genome phylogenetic analyses (Rudra and Gupta, 2024). However, multiple groups remain within the genus, such as the *P. fluorescens* group, which can further be divided into several subgroups, such as the *P. gessardii* subgroup (Lalucat et al., 2020; Lalucat et al., 2022). Species of the *P. gessardii* subgroup have been mostly described based on strains isolated from water samples, soil or plants, and strains isolated from fish (Duman et al., 2021). Although reports on infections are scattered, they are related to the *P. fluorescens* subgroup, which encompasses species that apart from multiple environments, have been isolated from clinical samples (Scales et al., 2014).

*Pseudomonas aeruginosa* and other clinically important *Pseudomonas* spp. are known to harbor natural and acquired resistance genes against different antibiotics, including β-lactams (Lodge et al., 1990; Girlich et al., 2004; Fajardo et al., 2014; Zhao and Hu, 2010). They exhibit resistance to several clinically important antimicrobials such as β-lactam antibiotics, including penicillin and cephalosporins, owing to the presence of AmpC β-lactamases and drug efflux pumps. Thus, infections caused by pathogenic species of the genus are often difficult to treat.

In this study, we used a polyphasic approach, including phylogenomic analyses, to confirm that three bacterial strains, previously isolated from gut samples of wild Atlantic mackerel (*Scomber scombrus*) from the northern North Sea (Marathe et al., 2022; Nimje and Marathe, 2023), represent a novel species within the *P. gessardii* subgroup of the genus *Pseudomonas*. The name *Pseudomonas imrae* sp. nov. is proposed. The strains exhibited high minimum inhibitory concentrations for several antibiotics and carry a gene encoding a novel variant of class C β-lactamase, *bla*_PFL-7_.

## Materials and methods

### Strain isolation and identification

Strains 16FHM2^T^ (=CCUG 74779^T^=CECT 30571^T^), 15FMM2 (=CCUG 74780) and 15FMM3 (=CCUG 74781) were isolated from gut contents of two specimens of wild Atlantic mackerel (*Scomber scombrus*) collected in the northern North Sea (International Council for the Exploration of the Sea, ICES region 4.a, in November 2018) (Marathe et al., 2022). The three strains were isolated on Mueller-Hinton (MH) agar containing meropenem (0.125 μg mL^−1^), incubated at 30°C, for 36 h.

Initial identification was performed, using matrix-assisted laser desorption ionization–time of flight mass spectrometry (MALDI-TOF MS), using Bruker MALDI Biotyper^®^ (Bruker Daltonics, Bremen, Germany), as previously described (Grevskott et al., 2024); the strains were identified as belonging to the genus *Pseudomonas* but could not be assigned to any described species. Total genomic DNA was extracted from strain 16FHM2^T^, using the previously described “heat-shock” protocol (Welinder-Olsson et al., 2000). The nearly complete 16S rRNA gene was amplified by PCR, using modified versions of primers (16F28, 5’-AGAGTTTGATCKTGGCTCAG-3′ and 16R1494, 5’-TACGGYTA CCTTGTTACGAC-3′) (Lane, 1991; Hauben et al., 1997), as described previously (Carvalheira et al., 2020). The PCR products were purified and sequenced, as described previously (Jaén-Luchoro et al., 2020), using modified versions sequencing primers (16F530, 5’-TTCGTGCCAGCAG CCGCGG-3′ 16R806, 5’-GGACTACCAGGGTATCTAAT-3′; 16F1103, 5’-TGTTGGGTTAAGTCCCGCAAC-3′, and 16R1494 5’-TACGGYTA CCTTGTTACGAC-3′) (Lane, 1991; Hauben et al., 1997; Buchholz-Cleven et al., 1997) and an Applied Biosystems SeqStudio 8 Flex Genetic Analyzer system (Thermo Fisher Scientific, Waltham, MA, United States). The partial *rpoD* sequence was determined, using previously-described primers and protocols for amplification and sequencing (Mulet et al., 2009). The 16S rRNA and *rpoD* gene sequences were analyzed using EzBiocloud (Yoon et al., 2017), and NCBI BLAST (Altschul et al., 1990) against sequences of type material of the Nucleotide collection (nr/nt) of GenBank (Sayers et al., 2024), respectively.

### Growth assays and biochemical tests

The strains were grown overnight on MH Agar medium and were characterized by biochemical profiling, using the CCUG NFX worksheet,^2^ including API ZYM and API 20NE commercial panels (bioMérieux, Marcy-l’Étoile, France). Bacterial growth was evaluated by streaking the strains on MH Agar plates and incubating at different temperatures ranging from 4 to 42°C for as long as 96 h.

### Microscopy analyses

Cell sizes and morphology were examined after 1 day incubation on Blood Agar medium, at 30°C, using a digital holotomographic microscope (DHM; HT-2, Tomocube Inc., Daejeon, South Korea), following previously described procedures (Fernández-Juárez et al., 2023). This enables non-invasive, label-free 3D imaging of bacterial cell morphology, based on obtaining tomographic refractive index (RI) data, which can subsequently be segmented to highlight particular cell structures based on their distinct RI signatures. For holotomographic imaging, bacterial colonies were recovered from the agar medium and re-suspended in phosphate-buffered saline (PBS). Then, 30 μL of the bacterial suspension was placed in a Tomodish (Tomocube Inc., Daejeon, South Korea) and covered with a coverslip in preparation for imaging. The resulting tomographic imaging data were visualized and analyzed, using TomoStudio and TomoAnalysis software (Tomocube Inc., Daejeon, South Korea). An average of 20 cells were imaged and analyzed, and the resulting images were exported to ImageJ software for additional analysis and figure preparation.

### Antimicrobial susceptibility testing

The strains were grown overnight on MH Agar medium with ampicillin (100 μg mL^−1^) and analyzed for antimicrobial sensitivity, using broth dilution method on Sensititre™ plates (Thermo Fisher Scientific, Waltham, MA, United States), following the manufacturer’s instructions, as described previously (Grevskott et al., 2024). Briefly, suspensions were prepared from freshly grown cultures to an optical density of 0.5 McFarland. Ten microliters of suspensions were added to 10 mL of MH Broth and added to sensititre plates. The plates were incubated at 30°C (optimal temperature for the strains) for 24 h and read manually. Minimum inhibitory concentrations (MICs) were determined as the lowest tested antimicrobial concentrations with no observed growth, as recommended by the EUCAST reading guide for broth microdilution (Version 5.0, January 2024).

### Cell fatty acid-fatty acid methyl ester analysis

The strains were cultivated on Trypticase Soy Agar medium at 28°C for 24 h for cellular fatty acid-fatty acid methyl ester (CFA-FAME) analysis. The CFA-FAME profiles were determined, using a gas chromatograph (HP 5890; Hewlett-Packard, Palo Alto, CA, United States), following a standardized protocol similar to that of the MIDI Sherlock MIS system (Sasser, 2001), as described previously (Zamora et al., 2012).

### Whole genome sequencing and genome assembly

Strains were cultivated on MH Agar medium with ampicillin 100 μg mL^−1^ at 30°C for 24 h, and genomic DNA was prepared, using a DNeasy Blood & Tissue kit (Qiagen, Hilden, Germany), for Illumina short-read sequencing, and a modified version (Salvà-Serra et al., 2018) of a previously described protocol (Marmur, 1961) for Oxford Nanopore long-read sequencing. For Illumina sequencing, a paired-end library was prepared, using a Nextera DNA Flex library prep kit, and sequenced on an Illumina MiSeq platform (Illumina, Inc., San Diego, CA, United States) at the Norwegian Sequencing Centre in Oslo. For Oxford Nanopore sequencing, a library was prepared, using a Rapid Barcoding Sequencing kit (SQK-RBK004), and sequenced for 72 h on a MinION device (Oxford Nanopore Technologies, Ltd., Oxford, United Kingdom), at the Research Lab of the Culture Collection University of Gothenburg (CCUG), using a FLO-MIN106 (version R9.4.1) flow cell and analyzed, using the software MinKNOW version 3.6.5 (Oxford Nanopore Technologies), with default parameters. The raw Oxford Nanopore reads were base-called, using Guppy version 3.4.5 and evaluated, using NanoPlot version 1.26.3 (De Coster et al., 2018). The Oxford Nanopore sequence reads were assembled *de novo* following a previously described protocol (Wick et al., 2023). Briefly, four subsets of the Oxford Nanopore reads were created, using Trycycler v0.5.5 (Wick et al., 2021). Each subset was assembled *de novo*, using Flye v2.9.5 (Kolmogorov et al., 2019), Raven v1.8.3 (Vaser and Šikić, 2021) and Canu v2.2 (Koren et al., 2017). A consensus assembly was generated, using Trycycler v0.5.5, and polished using three different methods: Medaka v1.11.3 (i.e., using the Oxford Nanopore reads)^3^, with the mode r941_min_high_g344; Polypolish v0.6.0 (i.e., using the Illumina reads) (Wick and Holt, 2022); and POLCA, using Masurca v4.1.0 (Zimin and Salzberg, 2020; Zimin et al., 2013). Manual observations of read mappings were performed, using UGENE v48.1 (Okonechnikov et al., 2012). The Illumina-only assemblies were performed, using SPAdes version v3.13 (Bankevich et al., 2012), and evaluated, using QUAST version 5.2 (Gurevich et al., 2013). The genome assemblies were annotated, using PGAP v6.3 and v6.9 (Tatusova et al., 2016).

### β-lactamase sequence analysis

The amino acid sequences of possible β-lactamases were analyzed, using TBLASTN against the entire Core nucleotide database (core_nt) of NCBI GenBank (Sayers et al., 2024). A second search was conducted, against the Nucleotide collection (nr/nt), restricting the search space to the genus *Pseudomonas* (Taxonomy ID: 286). Sequences were also analyzed using BLASTP against the Beta-Lactamase DataBase (Naas et al., 2017). The genetic context was analyzed, using Unipro UGENE v48.1 (Okonechnikov et al., 2012).

### Overall genome relatedness indices

Digital DNA–DNA hybridization (dDDH) (Auch et al., 2010) values were determined, using the Genome-to-Genome Distance Calculator (GGDC) v3.0 (Meier-Kolthoff et al., 2013). Average nucleotide identity values, based on BLAST (ANIb) (Goris et al., 2007; Altschul et al., 1990), were calculated, using the webserver JSpeciesWS (Richter et al., 2016). For each comparison, ANIb values were determined bi-directionally and the average calculated.

### Average nucleotide identity (ANI)-based dendrogram

The matrix containing the average ANIb values was used to generate a dendrogram, using the software PermutMatrix v1.9.3 (Caraux and Pinloche, 2005). The dendrogram was constructed, using Pearson’s distance correlation and hierarchical clustering with an average linkage method (UPGMA). The dendrogram was displayed, using the Interactive Tree of Life (iTOL) v7.1 (Letunic and Bork, 2024).

### Core genome-based phylogenomic analysis

A core genome-based phylogenomic tree was constructed, including type strains of species of the *P. fluorescens* and *P. gessardii* subgroups (Supplementary Table 1). Briefly, the genome sequences were annotated, using Prokka v1.14.6 (Seemann, 2014), and the annotated proteins sequences were compared, using the Software GET_HOMOLOGUES v17112020 (Contreras-Moreira and Vinuesa, 2013), with BLASTP (all vs. all) (Altschul et al., 1990). The sequences were clustered by applying a 70/70 criterion (i.e., 70% of identity over 70% of the length), using three clustering algorithms: BDBH; COGtriangles (Kristensen et al., 2010); and OrthoMCL (Li et al., 2003). The intersection of the three algorithms was used to determine a consensus core genome formed by single-copy orthologous sequences. The sequences were aligned, using Clustal Omega v1.2.0 (Sievers et al., 2011), and the alignment was trimmed, using Gblocks v0.91b (Castresana, 2000). Subsequently, the alignments were concatenated and a phylogenomic tree was constructed, using PhyML v20120412 (Guindon et al., 2010) and a Shimodaira-Hasegawa-like approximate likelihood-ratio test (SH-aLRT) for branching statistical support (Anisimova and Gascuel, 2006). The phylogenomic tree was visualized, using iTOL v7.1 (Letunic and Bork, 2024).

### Ecological distribution

Additional related strain genome sequences were searched by analyzing the partial *rpoD* sequence, using BLASTN (Altschul et al., 1990), against the Nucleotide collection (nt) of NCBI. Metagenome-assembled genomes (MAGs) of the proposed novel species were searched using Protologger v2 (Hitch et al., 2021). The Branchwater Metagenome Query platform (Irber et al., 2022) was used to search in more than one million metagenomic datasets from the Sequence Read Archive (SRA) (Leinonen et al., 2011).

## Results

### Strain isolation and characterization

During a screening for β-lactam-resistant bacteria in gut samples of wild Atlantic mackerel (*Scomber scombrus*) from the northern North Sea, three *Pseudomonas* spp. strains were isolated. The rod-shaped cells were Gram-stain-negative, forming smooth, translucent (2–3 mm wide) colonies when grown for 36 h on MH agar medium with ampicillin.

MALDI-TOF MS typing analysis could not identify the strains to the species level. The 16S rRNA gene sequence showed relatedness to *P. libanensis* (99.9%), as well as to *P. synxantha*, *P. gessardii* and *P. shahriarae* (99.9%) while partial *rpoD* sequence indicated distant relationships to *P. proteolytica* (93.4%) and *P. mucidolens* (92.7%) and, thus, could not be assigned to any existing species of the genus *Pseudomonas* (Girard et al., 2020). The discrepancies observed in the identifications by 16S rRNA gene and *rpoD* sequence analyses indicated a high probability that the strains represented a novel species.

**Figure 1:**
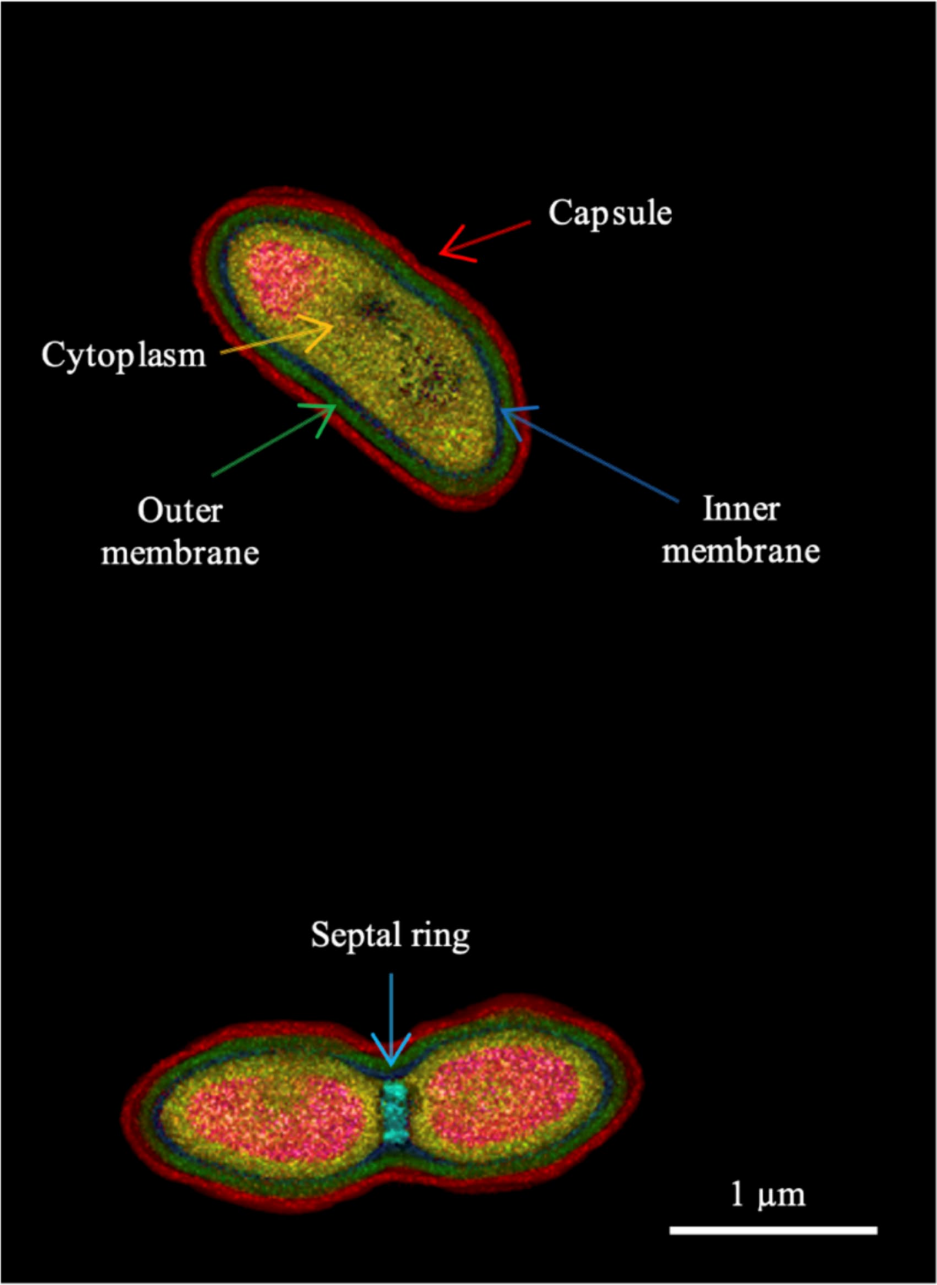
Holotomographic microscopy image of strain 16FHM2^T^ showing different morphological features of the cells, indicated by different color arrows. The lower part of the figure shows a dividing cell. The colors represent different refractive index ranges (see Supplementary Figure 1 for the refractive index scale). Strain 16FHM2^T^ was cultivated on Blood Agar medium, for 1 day, at 30°C.

The strains are Gram-negative and motile. The strains grow between 10 and 35°C but not at 4 or 42°C on MH Agar medium. Only one of the strains was able to grow when cultivated on blood agar at 37°C, suggesting that persistence in humans might be possible. The strains demonstrated good growth at salinities up to 3% (w/v) NaCl, while variable growth was observed at 4.5 and 5% NaCl. The strains are catalase- and oxidase-positive and exhibit gelatine hydrolysis, nitrate reduction and esterase activities. Additional phenotypic traits, including the results of multiple growth assays and biochemical analyses, are presented in Supplementary Table 2. The cell morphology was studied by digital holotomographic microscopy and the cell size was determined to be 1.6 ± 0.3 μm by 0.8 ± 0.05 μm (Figure 1).

### Antimicrobial susceptibility

The strains exhibited high MIC values for ampicillin (>64 μg mL^−1^), cephalosporins, such as cefazolin, cefuroxime and cefoxitin (>32 μg mL^−1^), and carbapenems, such as ertapenem (8 μg mL^−1^), as well as against azithromycin (8 μg mL^−1^) and trimethoprim (>16 μg mL^−1^), while the strains exhibited low MIC values for ciprofloxacin, gentamycin and tigecycline. The MICs for different antimicrobials tested are presented in Supplementary Table 3.

### Cell fatty acids

Strains 16FHM2^T^, 15FMM2 and 15FMM3 presented CFA-FAME profiles characteristic of species of the genus *Pseudomonas*: palmitic acid (C_16:0_), C_10:0_ 3-OH, C_12:0_ 2-OH and C_12:0_ 3-OH. The major CFAs of the strains were C_16:1_ ω7c (36.0–37.2%), C_16:0_ (21.6–23.4%), followed by the summed feature formed by C_18:1_ω7c, 12 t and/or 9 t (11.7–12.9%) and C_17:0_ cyclo (10.9–11.8%) (Table 1). Compared with *P. mucidolens* CCUG 1424^T^, the three strains present similar CFA-FAME patterns overall, although they displayed higher proportions of C_17:0_ cyclo and lower proportions of C_18:1_ω7c/12t/9t.

**Table 1:** Cellular fatty acid compositions (%) of the three strains of the proposed novel species, *P. imrae* sp. nov. and the type strain of *P. mucidolens*, its most closely-related species.

Fatty acid		ECL	Strain			*P. mucidolens* CCUG 1424^T^
			CCUG 74779^T^	CCUG 74780	CCUG 74781	
Saturated	C_12:0_	12.000	2.9	3.1	3.5	2.7
	C_16:0_	16.000	23.4	22.5	21.6	19.1
	C_17:0_	17.000	tr	tr	tr	tr
	C_18:0_	18.000	1.3	1.2	1	2
Hydroxy	C_10:0_ 3-OH	11.423	3.4	3.5	3.8	3.8
	C_12:0_ 2-OH	13.178	4.7	4.8	5.6	4.3
	C_12:0_ 3-OH	13.455	3.6	3.7	3.9	4.1
Unsaturated	C_16:1_ ω7c	15.819	36	37.2	37	38.7
	*Summed feature 7	17.824	12.9	12.5	11.7	18.4
Cyclopropane	C_17:0_ cyclo	16.888	11.2	10.9	11.8	6.5

*Summed feature 7 (contains C1_8:1_ω7c, C_18:1_ω9t and/or C_18:1_ω12t).

Tr, trace amounts (<1%).

ECL, equivalent chain length.

### Whole-genome sequencing and overall genome relatedness indices

The genomes of the three strains were sequenced, using an Illumina MiSeq platform. Additionally, strain 16FHM2^T^ was sequenced, using also an Oxford Nanopore MinION device. The assembly of strain 16FHM2^T^ resulted in a single complete sequence of 5,444,440 bp (Table 2). The assemblies of strains 15FMM2 and 15FMM3 resulted in two draft genome sequences of 5,406,388 and 5,405,281 bp, respectively. The number of coding sequences per genome ranged from 4,904 to 4,906 and the GC contents of the genomes were determined to be 58.9%.

**Table 2:** Genome features of the three strains of the proposed novel species, *P. imrae* sp. nov.

		*Strains*		
Section	Features	16FHM2^T^	15FMM2	15FMM3
Sequencing and assembly	Sequencing platforms	Illumina MiSeq + Oxford Nanopore MinION	Illumina MiSeq	Illumina MiSeq
	Assembly method	Flye v2.9.5, Raven v1.8.3, Canu v2.2, Trycycler v0.5.5	SPAdes v3.13	SPAdes v3.13
	Assembly coverage	96 X (Illumina) + 286 X (Oxford Nanopore)	110 X	59 X
	GenBank accession number	CP110853	JAPEQY000000000	JAPEQX000000000
	SRA accession numbers	SRR23726382 and SRR23770311	SRR23725248	SRR23725247
	Finishing quality	Complete genome	Draft genome	Draft genome
	Number of contigs	1	47	49
	Total length (bp)	5,444,440	5,406,388	5,405,281
	N50 (bp)	5,444,440	249,933	284,119
	Number of N’s	0	0	0
	GC content (%)	58.9	58.9	58.9
Annotation	Annotation method	PGAP v6.9	PGAP v6.3	PGAP v6.3
	Number of genes (total)	4,993	4,979	4,981
	Total coding sequences (CDS)	4,906	4,904	4,906
	Protein coding sequences	4,811	4,820	4,821
	Pseudogenes	95	84	85
	tRNA	67	61	61
	Non-coding RNA	4	4	4
	Ribosomal RNA	16 (5 operons)	4	4
	Hypothetical proteins	356	393	392

ANIb and dDDH were calculated between the three strains of the proposed novel species, and between strain 16FHM2^T^ and the type strains of 55 closely-related *Pseudomonas* species belonging to the *P. gessardii* and *P. fluorescens* subgroups of the *P. fluorescens* group. The ANIb and the dDDH values between the three strains of the proposed novel species were 99.99 and 100%, respectively, confirming that the three strains are very similar and closely related to each other. The ANIb values between the genome sequence of strain 16FHM2^T^ and those of the type strains of species of the *P. fluorescens* and *P. gessardii* subgroups with validly published names ranged from 87.4 to 82.2%, while the dDDH values ranged from 35.3 to 26.8% (Table 3). The analyses confirmed that the most closely related species is *P. mucidolens*. These data indicate that strain 16FHM2^T^ represents a novel species within the *P. gessardii* subgroup of the genus *Pseudomonas*.

**Table 3:** ANIb and dDDH values determined between the genome sequence of strain 16FHM2^T^ and the genome sequences of the type strains of species of the *P. gessardii* and *P. fluorescens* subgroups.

Strain	dDDH (%)	ANIb (%)
*Pseudomonas mucidolens* LMG 2223^T^	35.3	87.38
*Pseudomonas shahriarae* SWRI52^T^	30.7	84.91
*Pseudomonas brenneri* DSM 15294^T^	30.6	84.88
*Pseudomonas proteolytica* LMG 22710^T^	30.8	84.84
*Pseudomonas gessardii* LMG 21604^T^	30.7	84.75
*Pseudomonas karstica* CCM 7891^T^	28.5	83.51
*Pseudomonas spelaei* CCM 7893^T^	28.5	83.50
*Pseudomonas yamanorum* LMG 27247^T^	28.8	83.32
*Pseudomonas fildesensis* KG01^T^	28.2	83.16
*Pseudomonas grimontii* DSM 17515^T^	28.3	83.05
*Pseudomonas veronii* DSM 11331^T^	28.3	82.97
*Pseudomonas panacis* DSM 18529^T^	28.1	82.96
*Pseudomonas marginalis* NCPPB 667^T^	28.3	82.95
*Pseudomonas pergaminensis* 1008^T^	28.0	82.91
*Pseudomonas extremaustralis* DSM 17835^T^	28.2	82.85
*Pseudomonas allii* MAFF 301514^T^	28.0	82.82
*Pseudomonas aylmerensis* S1E40^T^	28.3	82.81
*Pseudomonas azotoformans* LMG 21611^T^	28.1	82.81
*Pseudomonas lurida* LMG 21995^T^	27.8	82.81
*Pseudomonas extremorientalis* CCUG 51517^T^	27.8	82.80
*Pseudomonas libanensis* DSM 17149^T^	27.5	82.79
*Pseudomonas petroselini* MAFF 311094^T^	27.7	82.79
*Pseudomonas canadensis* 2-92^T^	27.7	82.79
*Pseudomonas salmasensis* SWRI126^T^	27.9	82.78
*Pseudomonas haemolytica* DSM 108987^T^	27.5	82.78
*Pseudomonas lactucae* MAFF 301380^T^	27.8	82.78
*Pseudomonas asgharzadehiana* SWRI132^T^	27.6	82.76
*Pseudomonas fluorescens* ATCC 13525^T^	27.8	82.75
*Pseudomonas azadiae* SWRI103^T^	28.1	82.74
*Pseudomonas khavaziana* SWRI124^T^	27.4	82.74
*Pseudomonas paracarnis* V5/DAB/2/5^T^	27.5	82.74
*Pseudomonas antarctica* LMG 22709^T^	27.7	82.73
*Pseudomonas edaphica* RD25^T^	28.2	82.72
*Pseudomonas simiae* CCUG 50988^T^	27.4	82.72
*Pseudomonas lactis* DSM 29167^T^	27.8	82.67
*Pseudomonas carnis* B4-1^T^	27.8	82.67
*Pseudomonas salomonii* ICMP 14252^T^	27.6	82.65
*Pseudomonas tritici* SWRI145^T^	27.6	82.64
*Pseudomonas cyclaminis* MAFF 301449^T^	27.9	82.64
*Pseudomonas paralactis* DSM 29164^T^	27.7	82.64
*Pseudomonas cedrina* LMG 23661^T^	28.2	82.63
*Pseudomonas orientalis* LMG 23660^T^	27.9	82.63
*Pseudomonas synxantha* NCTC 10696^T^	27.5	82.57
*Pseudomonas cremori*s WS 5106^T^	27.5	82.55
*Pseudomonas costantinii* LMG 22119^T^	27.7	82.54
*Pseudomonas trivialis* DSM 14937^T^	27.5	82.54
*Pseudomonas palleroniana* LMG 23076^T^	27.5	82.52
*Pseudomonas tolaasii* NCPPB 2192^T^	27.8	82.51
*Pseudomonas nabeulensis* E10B^T^	27.8	82.48
*Pseudomonas sivasensis* P7^T^	27.3	82.45
*Pseudomonas kairouanensis* KC12^T^	27.7	82.45
*Pseudomonas pisciculturae* P115^T^	27.5	82.41
*Pseudomonas poae* LMG 21465^T^	27.6	82.38
*Pseudomonas rhodesiae* DSM 14020^T^	26.8	82.18
*Pseudomonas kitaguniensis* MAFF 212408^T^	27.1	82.16

### Whole-genome sequence ANIb dendrogram

**Figure 2:**
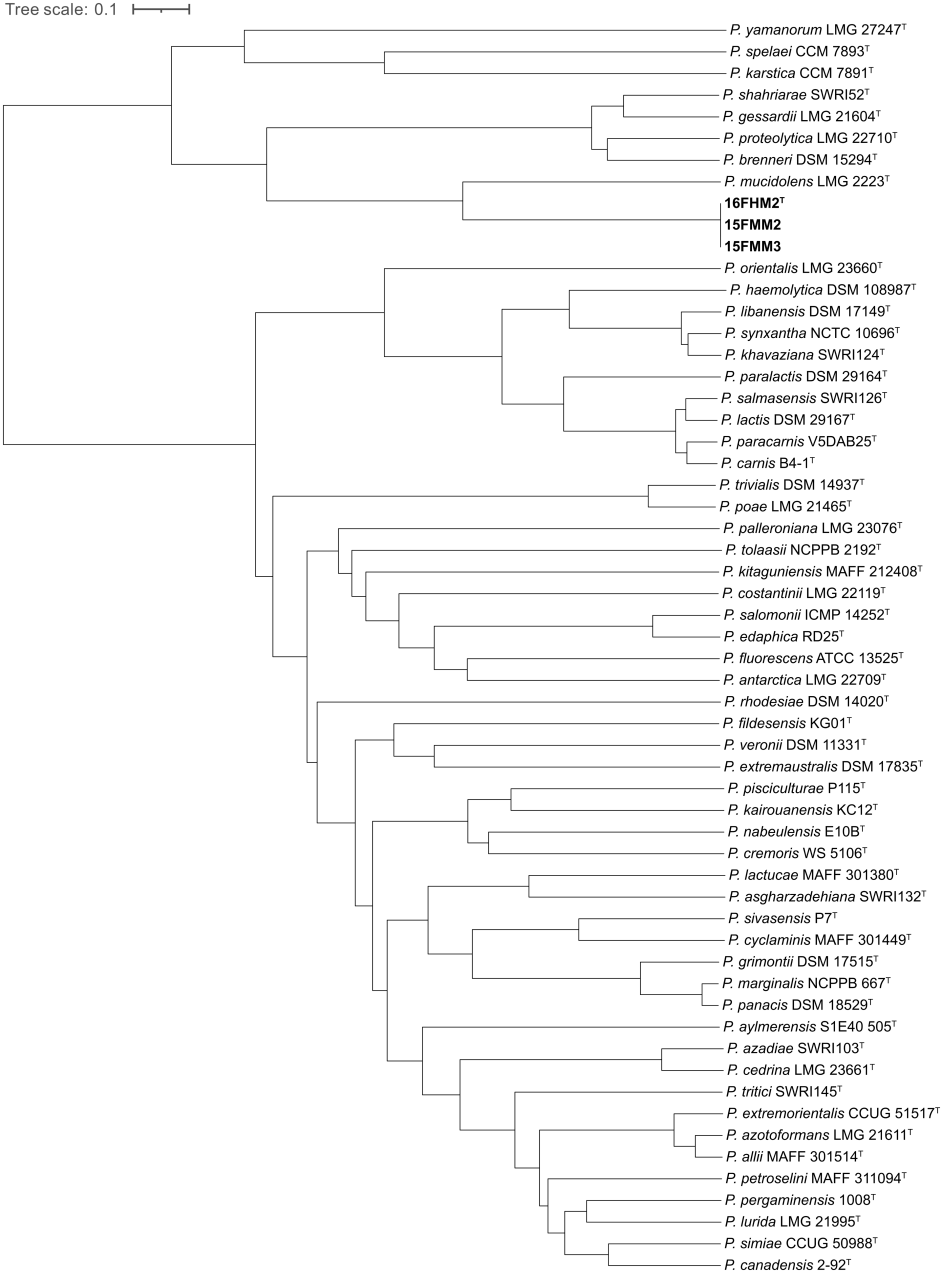
Dendrogram generated from whole-genome sequence ANIb determinations, demonstrating the estimated relationships of the three strains of the proposed novel species and type strains of species of the *P. gessardii* and *P. fluorescens* subgroups.

The ANIb values were determined between (all vs. all) genome sequences of type strains of the species of the *P. gessardii* and *P. fluorescens* subgroups. The values ranged from 82.02% (between *P. mucidolens* and *P. kitaguniensis*) to 96.44% (between *P. panacis* and *P. marginalis*). The ANIb values of strain 16FHM2^T^ compared with the type strains of other species ranged from 87.34% (*P. mucidolens* LMG 2223^T^) and 82.16% (*P. kitaguniensis* MAFF 212408^T^), which suggests that *P. mucidolens* is the most closely related species. Indeed, the dendrogram also shows the relationship of *P. mucidolens* to the proposed novel species, within the cluster formed by species of the *P. gessardii* subgroup (Figure 2).

### Core genome-based phylogenomic analysis

**Figure 3:**
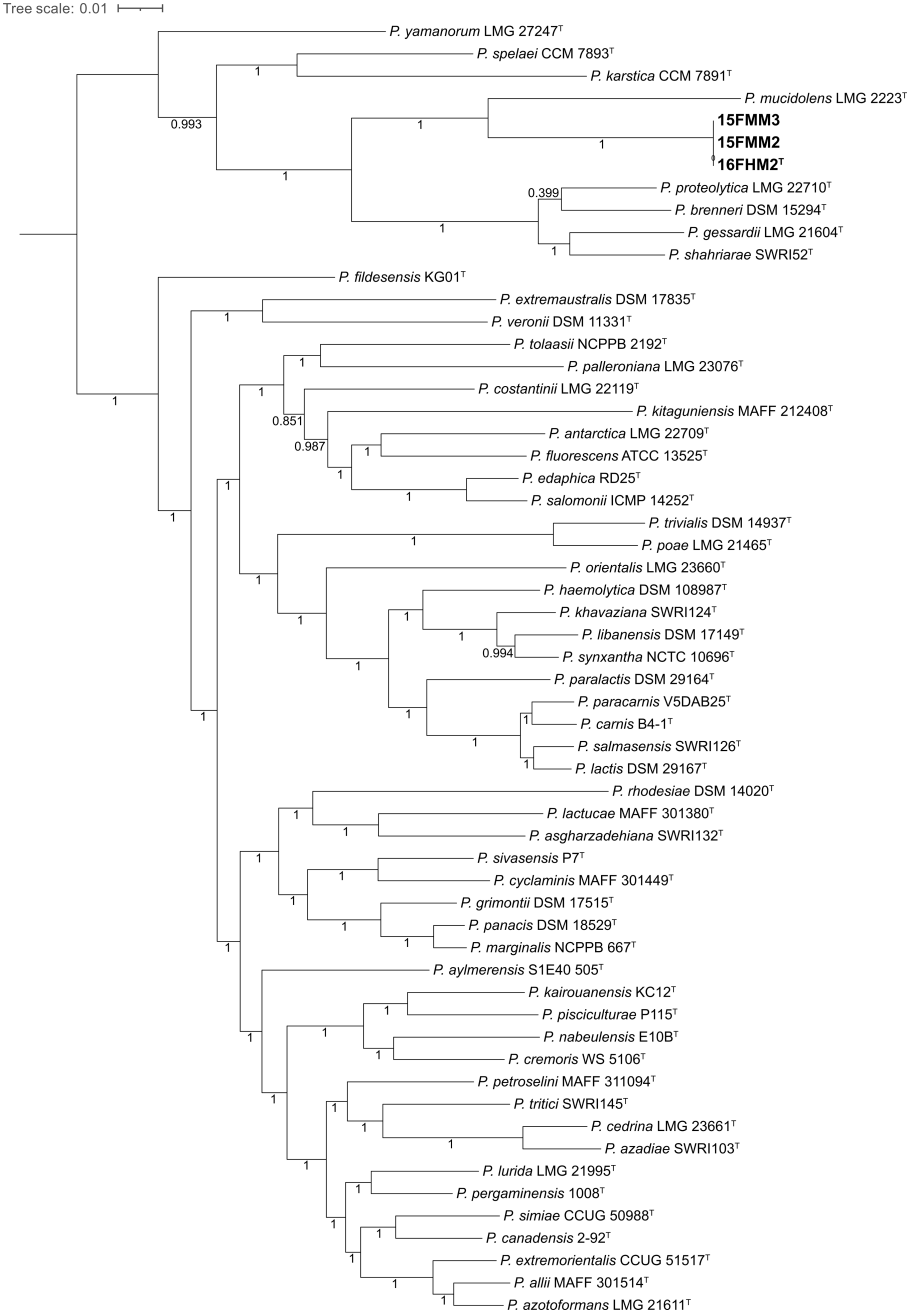
Core genome-based phylogenomic tree of the three strains of the proposed novel species and type strains of species of the *P. gessardii* and *P. fluorescens* subgroups. The tree was constructed, using Maximum Likelihood and the Shimodaira-Hasegawa-like approximate likelihood-ratio test (SH-aLRT). The numbers at the nodes indicate the SH-aLRT support values.

A total of 381,851 amino acid positions, encoded by 1,361 single-copy shared genes, were used to construct the core genome-based phylogenomic tree, including the genome sequences of the type strains of species of the *P. gessardii* and *P. fluorescens* subgroups. The core genome confirms that the species are divided in two well-defined clusters, corresponding to the two subgroups included in the analysis, and that the proposed novel species is a member of the *P. gessardii* subgroup. Additionally, the analysis confirms that *P. mucidolens* is the most closely related species (Figure 3).

### Class C β-lactamase

We detected a new variant of class C β-lactamase in our strains (NCBI Reference Sequence accession number: WP_410017811.1). This variant showed 85.3% amino acid identity (query coverage 100%) to a Class C β-lactamase from *P. mucidolens*. The search against the Beta-Lactamase DataBase revealed that it belongs to the family PFL and therefore it was designated PFL-7. The most closely related listed variant was PFL-5 (WP_017475175.1), from *Pseudomonas* sp. PAMC 26793, with 76% of amino acid identity. PFL-7 has a LysR family regulator (WP_410017810.1) encoded upstream of its gene, which shows 92.7% amino acid identity to the regulator from *P. mucidolens.* Thus, PFL-7 forms an AMP-C-AMP-R system detected in many bacteria (Balasubramanian et al., 2012).

### Ecological distribution

The *rpoD* sequence-based search of the Nucleotide collection (nt) of NCBI did not detect any additional strain of the proposed novel species. No MAGs of the proposed novel species were found when screening thousands of MAGs, using Protologger. The Branchwater Metagenome Query search did not yield any match with a containment ANI (cANI) score larger than 0.97, which often represents a species-level match, but yielded 41 matches with a cANI score between 0.95 (33 matches) and 0.96 (8 matches) (Supplementary Table 4). These matches probably do not represent members of the same species but might represent related strains of related taxa within the analyzed metagenomic datasets. The samples originated from fresh water (*n* = 13), wastewater (*n* = 21) and food samples (leafy greens, chicken and a meat factory) (*n* = 7).

## Discussion

Three β-lactam-resistant isolates were obtained from gut samples of wild Atlantic mackerel (*Scomber scombrus*) from the northern North Sea and could not be assigned to any previously described species. Using a polyphasic approach, including phenotypic, chemotaxonomic, phylogenetic and phylogenomic analyses, we have demonstrated that these three isolates represent a novel species of *Pseudomonas* within the *P. gessardii* subgroup of the *P. fluorescens* group, for which the name *Pseudomonas imrae* sp. nov. is proposed. The species belonging to the genus *Pseudomonas* and, more particularly, species of the *P. gessardii* subgroup, are widespread in aquatic environments, including marine habitats. *P. proteolytica* was isolated from water bodies in Antarctica (Reddy et al., 2004), *P. gessardii* and *P. brenneri* from natural mineral waters (Baida et al., 2001), *P. yamanorum* from soil on the coast of Observatorio island in south Patagonia (subantarctic environment), *P. karstica* and *P. spelaei* from caves (Arnau et al., 2015; Švec et al., 2020), and additional strains of species of the subgroup were isolated from fish (Duman et al., 2021). These observations are in accordance with the results of the Branchwater Metagenome Query, in which 33 of the 41 metagenomic samples containing related strains originated from fresh water or wastewater. This suggests that subgroup-specific adaptations for aquatic environments might be observed in the *P. gessardii* subgroup, although some species were found that were associated with plants and animals, such as *P. mucidolens* (isolated from egg) (Levine and Anderson, 1932) or *P. shahriarae*, isolated from rhizosphere of wheat (Girard et al., 2021).

*Pseudomonas* has a complex taxonomy with several species showing high similarities in biochemical analyses, as well as in studies using molecular markers, such as the 16S rRNA gene sequence. To overcome difficulties in classifying and differentiating *Pseudomonas* species, using such methods, alternative protocols, such as multilocus sequence analysis (MLSA) and whole genome sequence analysis have been proposed (Gomila et al., 2015). MLSA, using gene sequences, such as *gyrB*, *rpoB* and *rpoD*, and whole genome sequence analysis, using parameters, such as ANIb and core genome-based phylogeny, have made it possible to resolve the taxonomic spectrum of the genus *Pseudomonas*. Using these tools, several species have been recently reclassified (Lalucat et al., 2022; Rudra and Gupta, 2024). Our study adds to the recognition of the diversity of *Pseudomonas* species and highlights the use of for whole genome sequence analysis for the definitive resolution of the taxonomy of genus *Pseudomonas*. We further determined the complete genome sequence of the type strain of the proposed novel species. Using the described polyphasic approach, we have demonstrated that the three strains isolated and characterized in our study represent a novel species in the *P. gessardii* subgroup of *Pseudomonas*, for which the name *Pseudomonas imrae* sp. nov. is proposed.

*Pseudomonas* spp. are known to be intrinsically resistant to several antimicrobials including commonly used antibiotics and disinfectants (Poole, 2011). PFL-7 forms a typical AmpC-AmpR system detected in many bacteria (Balasubramanian et al., 2012). Amp-R is a regulator that modulates *ampC* expression. High expression of *ampC*, due to higher expression of *ampR*, has been shown in previous studies to be associated with resistance against a variety of β-lactam antibiotics, including penicillins, cephalosporins and, sometimes, carbapenems (Tariq et al., 2023). Along with activation of AmpC, AmpR also regulates genes responsible for recycling of cell wall/peptidoglycan on stimuli of cell wall damage, thus, emphasizing the presence of this system in a variety of bacteria including the genus *Pseudomonas* (Gyger et al., 2024; Balasubramanian et al., 2015). The presence of AmpC-AmpR system in our strain, may thus, explain the high MIC observed for *P. imrae* against different β-lactam antibiotics.

### Description of *Pseudomonas imrae* sp. nov.

*Pseudomonas imrae* (im’rae. N.L. gen. n. *imrae*, formed from IMR, acronym for Institute of Marine Research, Norway, where the first strains were isolated and studied).

Cells are Gram-negative, rod shaped, non-spore forming, and strictly aerobic, positive for catalase and oxidase. Optimum temperature for growth is 25–30°C, with no growth observed at 4°C and 42°C. The strains are positive for catalase, oxidase, acid phosphatase, esterase, gelatin hydrolysis, l-Arginine dihydrolase activity, and nitrate reduction, while they are negative for urease, *α*-fucosidase activity, α-mannosidase activity, *N*-acetyl-β-glucosaminidase activity, β-glucosidase activity, α-glucosidase activity, β-glucuronidase activity, β-galactosidase, α-galactosidase, acetamide utilization, DNAse activity, indole production and esculin hydrolysis. The strains can grow in the presence of 3% NaCl and show variable growth at 4.5 and 5% NaCl with no growth observed above 6% NaCl. The predominant cell fatty acids are C_16:1_ ω7c and C_16:0_, followed by C_18:1_ω7c/12 t/9 t and C_17:0_ cyclo, which are present in lower levels.

The 16S rRNA gene sequence is highly similar to *P. libanensis*, *P. synxantha*, *P. gessardii* and *P. shahriarae* (99.9%), while the partial *rpoD* sequence shows highest similarity to *P. proteolytica* (93.4%). Members belong to class *Gammaproteobacteria*, order *Pseudomonadales*, family *Pseudomonadaceae,* genus *Pseudomonas*, *P. fluorescens* group, *P. gessardii* subgroup. The type strain of the species is *Pseudomonas imrae* strain 16FHM2^T^ (=CCUG 74779^T^ = CECT 30571^T^); strains15FMM2 (=CCUG 74780) and 15FMM3 (= CCUG 74781) are other representatives. The strains were isolated from gut contents of two specimens of wild Atlantic mackerel (*Scomber scombrus*) collected in the northern North Sea, ICES region 4.a, in November 2018.

## Conclusion

Using a combination of phenotyping methods, genomics and phylogenomics, *Pseudomonas imrae* sp. nov. is described as a novel species of the genus *Pseudomonas*, belonging to the *P. gessardii* subgroup of the *P. fluorescens* group, isolated from the gut contents of Atlantic mackerel in Norway. The three characterized strains of *P. imrae* carry a novel class C β-lactamase gene variant. Our study highlights the importance of whole genome sequencing in bacterial taxonomy.

## Data Availability

The strains are deposited and available at the Culture Collection University of Gothenburg (CCUG) under the accession numbers CCUG 74779^T^ (=16FHM2^T^), CCUG 74780 (=15FMM2) and CCUG 74781 (=15FMM3). The type strain is also deposited and available at the Spanish Type Culture Collection (CECT, Valencia, Spain) under the accession number CECT 30571^T^. The genome sequences of the strains CCUG 74779^T^ (=16FHM2^T^), CCUG 74780 (=15FMM2) and CCUG 74781 (=15FMM3) have been deposited in DDBJ/ENA/GenBank under the accession numbers CP110853, JAPEQY000000000 and JAPEQX000000000, respectively. The Illumina and the Oxford Nanopore sequence reads are deposited and publicly available at the Sequence Read Archive (SRA) under the accession numbers SRR23726382, SRR23770311, SRR23725248 and SRR23725247. The nearly-complete 16S rRNA gene sequence and the partial *rpoD* gene sequence for strain 16FHM2^T^, determined by Sanger sequencing, are deposited in DDBJ/ENA/GenBank under the accession numbers PQ479520 and PQ505025, respectively.

## Footnotes

^1^ https://lpsn.dsmz.de/genus/pseudomonas

^2^ https://ccug.se/documents/worksheets/nfx.pdf

^3^ https://github.com/nanoporetech/medaka

## Supplementary material

SUPPLEMENTARY FIGURE 1 Reconstruction of *Pseudomonas imrae* strain 16FHM2^T^ using its refractive index, obtained with holotomographic imaging.

SUPPLEMENTARY TABLE 1 List of genome sequences of type strains of species of the *Pseudomonas gessardii* and *Pseudomonas fluorescens* subgroups included in the study.

SUPPLEMENTARY TABLE 2 Phenotypic characteristics of the three strains of the proposed novel species and the type strain of *Pseudomonas mucidolens*.

SUPPLEMENTARY TABLE 3 Minimum Inhibitory Concentrations (MIC) of different antimicrobials determined using Sensititre^TM^ Standard AST Plates (Thermo Scientific, United States) for the three strains of the proposed novel species.

SUPPLEMENTARY TABLE 4 Top matches from the search of strain 16FHM2^T^ using the Branchwater Metagenome Query platform.

## References

[ref1] Altschul S. F. Gish W. Miller W. Myers E. W. Lipman D. J. (1990). Basic local alignment search tool. J. Mol. Biol. 215, 403–410. doi: 10.1016/S0022-2836(05)80360-2, PMID: 2231712

[ref2] Anisimova M. Gascuel O. (2006). Approximate likelihood-ratio test for branches: a fast, accurate, and powerful alternative. Syst. Biol. 55, 539–552. doi: 10.1080/10635150600755453, PMID: 16785212

[ref3] Arnau V. G. Sánchez L. A. Delgado O. D. (2015). *Pseudomonas yamanorum* sp. nov., a psychrotolerant bacterium isolated from a subantarctic environment. Int. J. Syst. Evol. Microbiol. 65, 424–431. doi: 10.1099/ijs.0.065201-0 25385990

[ref4] Auch A. F. Von Jan M. Klenk H.-P. Göker M. (2010). Digital Dna-Dna hybridization for microbial species delineation by means of genome-to-genome sequence comparison. Stand. Genomic Sci. 2, 117–134. doi: 10.4056/sigs.531120, PMID: 21304684 PMC3035253

[ref5] Baida N. Yazourh A. Singer E. Izard D. (2001). *Pseudomonas brenneri* sp. nov., a new species isolated from natural mineral waters. Res. Microbiol. 152, 493–502. doi: 10.1016/S0923-2508(01)01223-2 11446518

[ref6] Balasubramanian D. Kumari H. Mathee K. (2015). *Pseudomonas aeruginosa* AmpR: an acute–chronic switch regulator. Pathogens Dis. 73, 1–14. doi: 10.1111/2049-632X.12208 PMC4542883 25066236

[ref7] Balasubramanian D. Schneper L. Merighi M. Smith R. Narasimhan G. Lory S.. (2012). The regulatory repertoire of *Pseudomonas aeruginosa* AmpC ß-lactamase regulator AmpR includes virulence genes. PLoS One 7:e34067. doi: 10.1371/journal.pone.0034067, PMID: 22479525 PMC3315558

[ref8] Bankevich A. Nurk S. Antipov D. Gurevich A. A. Dvorkin M. Kulikov A. S.. (2012). Spades: a new genome assembly algorithm and its applications to single-cell sequencing. J. Comput. Biol. 19, 455–477. doi: 10.1089/cmb.2012.0021, PMID: 22506599 PMC3342519

[ref9] Buchholz-Cleven B. E. E. Rattunde B. Straub K. L. (1997). Screening for genetic diversity of isolates of anaerobic Fe(ii)-oxidizing Bacteria using Dgge and whole-cell hybridization. Syst. Appl. Microbiol. 20, 301–309. doi: 10.1016/S0723-2020(97)80077-X

[ref10] Caraux G. Pinloche S. (2005). PermutMatrix: a graphical environment to arrange gene expression profiles in optimal linear order. Bioinformatics 21, 1280–1281. doi: 10.1093/bioinformatics/bti141 15546938

[ref11] Carvalheira A. Gonzales-Siles L. Salvà-Serra F. Lindgren Å. Svensson-Stadler L. Thorell K.. (2020). *Acinetobacter portensis* sp. nov. and *Acinetobacter guerrae* sp. nov., isolated from raw meat. Int. J. Syst. Evol. Microbiol. 70, 4544–4554. doi: 10.1099/ijsem.0.004311, PMID: 32618559

[ref12] Castresana J. (2000). Selection of conserved blocks from multiple alignments for their use in phylogenetic analysis. Mol. Biol. Evol. 17, 540–552. doi: 10.1093/oxfordjournals.molbev.a026334, PMID: 10742046

[ref13] Contreras-Moreira B. Vinuesa P. (2013). GET_HOMOLOGUES, a versatile software package for scalable and robust microbial pangenome analysis. Appl. Environ. Microbiol. 79, 7696–7701. doi: 10.1128/AEM.02411-13, PMID: 24096415 PMC3837814

[ref14] De Coster W. Dhert S. Schultz D. T. Cruts M. Van Broeckhoven C. (2018). NanoPack: visualizing and processing long read sequencing data. Bioinformatics 34, 2666–2669. doi: 10.1093/bioinformatics/bty149, PMID: 29547981 PMC6061794

[ref15] Dong X. Rao Z. Wu S. Peng F. Xie Z. Long Y. (2023). *Pseudomonas benzopyrenica* sp. nov., isolated from soil, exhibiting high-efficiency degradation of benzo(a)pyrene. Int. J. Syst. Evol. Microbiol. 73:6034. doi: 10.1099/ijsem.0.006034, PMID: 37725099

[ref16] Duman M. Mulet M. Altun S. Saticioglu I. B. Ozdemir B. Ajmi N.. (2021). The diversity of *Pseudomonas* species isolated from fish farms in Turkey. Aquaculture 535:736369. doi: 10.1016/j.aquaculture.2021.736369

[ref17] Fajardo A. Hernando-Amado S. Oliver A. Ball G. Filloux A. Martinez J. L. (2014). Characterization of a novel Zn2+−dependent intrinsic imipenemase from *Pseudomonas aeruginosa*. J. Antimicrob. Chemother. 69, 2972–2978. doi: 10.1093/jac/dku267, PMID: 25185138

[ref18] Fernández-Juárez V. Hallstrøm S. Pacherres C. O. Wang J. Coll-Garcia G. Kühl M.. (2023). Biofilm formation and cell plasticity drive diazotrophy in an anoxygenic phototrophic bacterium. Appl. Environ. Microbiol. 89, e01027–e01023. doi: 10.1128/aem.01027-23 37882569 PMC10686084

[ref19] Girard L. Lood C. Höfte M. Vandamme P. Rokni-Zadeh H. Van Noort V.. (2021). The ever-expanding *Pseudomonas* genus: description of 43 new species and partition of the *Pseudomonas putida* group. Microorganisms 9:1766. doi: 10.3390/microorganisms9081766, PMID: 34442845 PMC8401041

[ref20] Girard L. Lood C. Rokni-Zadeh H. Van Noort V. Lavigne R. De Mot R. (2020). Reliable identification of environmental *Pseudomonas* isolates using the *rpoD* gene. Microorganisms 8:1166. doi: 10.3390/microorganisms8081166 32752051 PMC7463772

[ref21] Girlich D. Naas T. Nordmann P. (2004). Biochemical characterization of the naturally occurring Oxacillinase Oxa-50 of *Pseudomonas aeruginosa*. Antimicrob. Agents Chemother. 48, 2043–2048. doi: 10.1128/AAC.48.6.2043-2048.2004, PMID: 15155197 PMC415580

[ref22] Gomila M. Peña A. Mulet M. Lalucat J. García-Valdés E. (2015). Phylogenomics and systematics in *Pseudomonas*. Front. Microbiol. 6:214. doi: 10.3389/fmicb.2015.00214 26074881 PMC4447124

[ref23] Goris J. Konstantinidis K. T. Klappenbach J. A. Coenye T. Vandamme P. Tiedje J. M. (2007). Dna-Dna hybridization values and their relationship to whole-genome sequence similarities. Int. J. Syst. Evol. Microbiol. 57, 81–91. doi: 10.1099/ijs.0.64483-0, PMID: 17220447

[ref24] Grevskott D. H. Radisic V. Salvà-Serra F. Moore E. R. B. Akervold K. S. Victor M. P.. (2024). Emergence and dissemination of epidemic-causing Oxa-244 carbapenemase-producing *Escherichia coli* St38 through hospital sewage in Norway, 2020–2022. J. Hosp. Infect. 145, 165–173. doi: 10.1016/j.jhin.2023.12.020, PMID: 38286237

[ref25] Guindon S. Dufayard J. F. Lefort V. Anisimova M. Hordijk W. Gascuel O. (2010). New algorithms and methods to estimate maximum-likelihood phylogenies: assessing the performance of Phyml 3.0. Syst. Biol. 59, 307–321. doi: 10.1093/sysbio/syq010, PMID: 20525638

[ref26] Gurevich A. Saveliev V. Vyahhi N. Tesler G. (2013). Quast: quality assessment tool for genome assemblies. Bioinformatics 29, 1072–1075. doi: 10.1093/bioinformatics/btt086, PMID: 23422339 PMC3624806

[ref27] Gyger J. Torrens G. Cava F. Bernhardt T. G. Fumeaux C. (2024). A potential space-making role in cell wall biogenesis for SltB1and DacB revealed by a beta-lactamase induction phenotype in *Pseudomonas aeruginosa*. MBio 15, e01419–e01424. doi: 10.1128/mbio.01419-24, PMID: 38920394 PMC11253642

[ref28] Hauben L. Vauterin L. Swings J. Moore E. R. (1997). Comparison of 16S ribosomal Dna sequences of all *Xanthomonas* species. Int. J. Syst. Bacteriol. 47, 328–335. doi: 10.1099/00207713-47-2-328, PMID: 9103617

[ref29] Hitch T. C. A. Riedel T. Oren A. Overmann J. Lawley T. D. Clavel T. (2021). Automated analysis of genomic sequences facilitates high-throughput and comprehensive description of bacteria. Isme Communications 1:16. doi: 10.1038/s43705-021-00017-z, PMID: 36732617 PMC9723785

[ref30] Ikuta K. S. Swetschinski L. R. Robles Aguilar G. Sharara F. Mestrovic T. Gray A. P.. (2022). Global mortality associated with 33 bacterial pathogens in 2019: a systematic analysis for the global burden of disease study 2019. Lancet 400, 2221–2248. doi: 10.1016/S0140-6736(22)02185-7, PMID: 36423648 PMC9763654

[ref31] Irber L. Pierce-Ward N. T. Brown C. T. (2022). Sourmash Branchwater enables lightweight petabyte-scale sequence search. [Epubh ahead of preprint]. doi: 10.1101/2022.11.02.514947

[ref32] Jaén-Luchoro D. Gonzales-Siles L. Karlsson R. Svensson-Stadler L. Molin K. Cardew S.. (2020). *Corynebacterium sanguinis* sp. nov., a clinical and environmental associated corynebacterium. Syst. Appl. Microbiol. 43:126039. doi: 10.1016/j.syapm.2019.126039 31776051

[ref33] Kolmogorov M. Yuan J. Lin Y. Pevzner P. A. (2019). Assembly of long, error-prone reads using repeat graphs. Nat. Biotechnol. 37, 540–546. doi: 10.1038/s41587-019-0072-8, PMID: 30936562

[ref34] Koren S. Walenz B. P. Berlin K. Miller J. R. Bergman N. H. Phillippy A. M. (2017). Canu: scalable and accurate long-read assembly via adaptive *k*-mer weighting and repeat separation. Genome Res. 27, 722–736. doi: 10.1101/gr.215087.116, PMID: 28298431 PMC5411767

[ref35] Kristensen D. M. Kannan L. Coleman M. K. Wolf Y. I. Sorokin A. Koonin E. V.. (2010). A low-polynomial algorithm for assembling clusters of orthologous groups from intergenomic symmetric best matches. Bioinformatics 26, 1481–1487. doi: 10.1093/bioinformatics/btq229, PMID: 20439257 PMC2881409

[ref36] Lalucat J. Gomila M. Mulet M. Zaruma A. García-Valdés E. (2022). Past, present and future of the boundaries of the *Pseudomonas* genus: proposal of *Stutzerimonas* gen. Nov. Syst. Appl. Microbiol. 45:126289. doi: 10.1016/j.syapm.2021.126289 34920232

[ref37] Lalucat J. Mulet M. Gomila M. García-Valdés E. (2020). Genomics in bacterial taxonomy: impact on the genus *Pseudomonas*. Genes 11:139. doi: 10.3390/genes11020139, PMID: 32013079 PMC7074058

[ref38] Lane D. J. (1991). “16S/23S rrna sequencing” in Nucleic acid techniques in bacterial systematics. eds. Stackebrandt E. G. Michael (New York: Wiley).

[ref39] Leinonen R. Sugawara H. Shumway M. International Nucleotide Sequence Database Collaboration (2011). The sequence read archive. Nucleic Acids Res. 39, D19–D21. doi: 10.1093/nar/gkq1019, PMID: 21062823 PMC3013647

[ref40] Letunic I. Bork P. (2024). Interactive tree of life (itol) v6: recent updates to the phylogenetic tree display and annotation tool. Nucleic Acids Res. 52, W78–W82. doi: 10.1093/nar/gkae268, PMID: 38613393 PMC11223838

[ref41] Levine M. Anderson D. Q. (1932). Two new species of Bacteria causing mustiness in eggs. J. Bacteriol. 23, 337–347. doi: 10.1128/jb.23.4.337-347.1932, PMID: 16559557 PMC533329

[ref42] Li L. Stoeckert C. J. Roos D. S. (2003). Orthomcl: identification of ortholog groups for eukaryotic genomes. Genome Res. 13, 2178–2189. doi: 10.1101/gr.1224503, PMID: 12952885 PMC403725

[ref43] Lodge J. M. Minchin S. D. Piddock L. J. V. Busby S. J. W. (1990). Cloning, sequencing and analysis of the structural gene and regulatory region of the *Pseudomonas aeruginosa* chromosomal *ampC β*-lactamase. Biochem. J. 272, 627–631, PMID: 2125210 10.1042/bj2720627 PMC1149754

[ref44] Marathe N. P. Salvà-Serra F. Nimje P. S. Moore E. R. B. (2022). Novel plasmid carrying mobile colistin resistance gene *mcr-4.3* and mercury resistance genes in *Shewanella baltica*: insights into mobilization of *mcr-4.3* in *Shewanella* species. Microbiol. Spectr. 10, e02037–e02022. doi: 10.1128/spectrum.02037-22 PMC9769806 36374025

[ref45] Marmur J. (1961). A procedure for the isolation of deoxyribonucleic acid from micro-organisms. J. Mol. Biol. 3:208-In1. doi: 10.1016/S0022-2836(61)80047-8

[ref46] Meier-Kolthoff J. P. Auch A. F. Klenk H.-P. Göker M. (2013). Genome sequence-based species delimitation with confidence intervals and improved distance functions. BMC Bioinformat. 14:60. doi: 10.1186/1471-2105-14-60, PMID: 23432962 PMC3665452

[ref47] Mulet M. Bennasar A. Lalucat J. Garcia-Valdes E. (2009). An *rpoD*-based Pcr procedure for the identification of *Pseudomonas* species and for their detection in environmental samples. Mol. Cell. Probes 23, 140–147. doi: 10.1016/j.mcp.2009.02.001, PMID: 19268522

[ref48] Naas T. Oueslati S. Bonnin R. A. Dabos M. L. Zavala A. Dortet L.. (2017). Beta-lactamase database (Bldb) – structure and function. J. Enzyme Inhib. Med. Chem. 32, 917–919. doi: 10.1080/14756366.2017.1344235, PMID: 28719998 PMC6445328

[ref49] Nimje P. S. Marathe N. P. (2023). Genome sequence of *Vibrio anguillarum* isolates carrying a novel class a β-lactamase Van-1: do migratory fish transport novel resistance factors? J. Glob. Antimicrob. Resist. 32, 152–154. doi: 10.1016/j.jgar.2022.10.017 36356852

[ref50] Okonechnikov K. Golosova O. Fursov M. Team, U (2012). Unipro Ugene: a unified bioinformatics toolkit. Bioinformatics 28, 1166–1167. doi: 10.1093/bioinformatics/bts091, PMID: 22368248 10.1093/bioinformatics/bts091

[ref51] Palleroni N. J. (2015). “Pseudomonas” in Bergey's manual of systematics of Archaea and Bacteria. eds. Trujillo M. E. Dedysh S. Devos P. Hedlund B. Kämpfer P. Rainey F. A.. doi: 10.1002/9781118960608.gbm01210

[ref52] Peix A. Ramírez-Bahena M. H. Velázquez E. (2018). The current status on the taxonomy of *Pseudomonas* revisited: an update. Infect. Genet. Evol. 57, 106–116. doi: 10.1016/j.meegid.2017.10.026, PMID: 29104095 10.1016/j.meegid.2017.10.026

[ref53] Pieterse C. M. J. Berendsen R. L. De Jonge R. Stringlis I. A. Van Dijken A. J. H. Van Pelt J. A.. (2021). *Pseudomonas simiae* Wcs417: star track of a model beneficial rhizobacterium. Plant Soil 461, 245–263. doi: 10.1007/s11104-020-04786-9

[ref54] Poole K. (2011). *Pseudomonas aeruginosa*: resistance to the max. Front. Microbiol. 2:65. doi: 10.3389/fmicb.2011.00065 21747788 PMC3128976

[ref55] Qin S. Xiao W. Zhou C. Pu Q. Deng X. Lan L.. (2022). *Pseudomonas aeruginosa*: pathogenesis, virulence factors, antibiotic resistance, interaction with host, technology advances and emerging therapeutics. Signal Transduct. Target. Ther. 7:199. doi: 10.1038/s41392-022-01056-1 35752612 PMC9233671

[ref56] Reddy G. S. N. Matsumoto G. I. Schumann P. Stackebrandt E. Shivaji S. (2004). Psychrophilic pseudomonads from Antarctica: *Pseudomonas antarctica* sp. nov., *Pseudomonas meridiana* sp. nov. and *Pseudomonas proteolytica* sp. nov. Int. J. Syst. Evol. Microbiol. 54, 713–719. doi: 10.1099/ijs.0.02827-0, PMID: 15143013

[ref57] Richter M. Rosselló-Móra R. Oliver Glöckner F. Peplies J. (2016). JSpeciesWS: a web server for prokaryotic species circumscription based on pairwise genome comparison. Bioinformatics 32, 929–931. doi: 10.1093/bioinformatics/btv681, PMID: 26576653 PMC5939971

[ref58] Rudra B. Gupta R. S. (2024). Phylogenomics studies and molecular markers reliably demarcate genus *Pseudomonas sensu stricto* and twelve other *Pseudomonadaceae* species clades representing novel and emended genera. Front. Microbiol. 14:3665. doi: 10.3389/fmicb.2023.1273665, PMID: 38249459 PMC10797017

[ref59] Salvà-Serra F. Svensson-Stadler L. Busquets A. Jaén-Luchoro D. Karlsson R. Moore R. B. E.. (2018). A protocol for extraction and purification of high-quality and quantity bacterial Dna applicable for genome sequencing: a modified version of the Marmur procedure. Protocol Exchange. doi: 10.1038/protex.2018.084

[ref60] Sasser M. (2001). “Identification of Bacteria by gas chromatography of cellular fatty acids” in Technical note #101. ed. Sasser M. (Newark, DE, USA: MIDI, Inc.).

[ref61] Sayers E. W. Beck J. Bolton E. E. Brister J. R. Chan J. Comeau D. C.. (2024). Database resources of the National Center for biotechnology information. Nucleic Acids Res. 52, D33–D43. doi: 10.1093/nar/gkad1044, PMID: 37994677 PMC10767890

[ref62] Scales B. S. Dickson R. P. Lipuma J. J. Huffnagle G. B. (2014). Microbiology, genomics, and clinical significance of the *Pseudomonas fluorescens* species complex, an unappreciated colonizer of humans. Clin. Microbiol. Rev. 27, 927–948. doi: 10.1128/CMR.00044-14, PMID: 25278578 PMC4187640

[ref63] Seemann T. (2014). Prokka: rapid prokaryotic genome annotation. Bioinformatics 30, 2068–2069. doi: 10.1093/bioinformatics/btu153, PMID: 24642063

[ref64] Sievers F. Wilm A. Dineen D. Gibson T. J. Karplus K. Li W.. (2011). Fast, scalable generation of high-quality protein multiple sequence alignments using Clustal omega. Mol. Syst. Biol. 7:539.doi: 10.1038/msb.2011.75 21988835 PMC3261699

[ref65] Švec P. Kosina M. Zeman M. Holochová P. Králová S. Němcová E.. (2020). *Pseudomonas karstica* sp. nov. and *Pseudomonas spelaei* sp. nov., isolated from calcite moonmilk deposits from caves. Int. J. Syst. Evol. Microbiol. 70, 5131–5140. doi: 10.1099/ijsem.0.004393, PMID: 32821035

[ref66] Tariq F. N. Shafiq M. Khawar N. Habib G. Gul H. Hayat A.. (2023). The functional repertoire of *AmpR* in the *AmpC* β-lactamase high expression and decreasing β-lactam and aminoglycosides resistance in Esbl *Citrobacter freundii*. Heliyon 9:e19486. doi: 10.1016/j.heliyon.2023.e19486, PMID: 37662790 PMC10472055

[ref67] Tatusova T. Dicuccio M. Badretdin A. Chetvernin V. Nawrocki E. P. Zaslavsky L.. (2016). Ncbi prokaryotic genome annotation pipeline. Nucleic Acids Res. 44, 6614–6624. doi: 10.1093/nar/gkw569, PMID: 27342282 PMC5001611

[ref68] Vaser R. Šikić M. (2021). Time- and memory-efficient genome assembly with raven. Nat. Comput. Sci. 1, 332–336. doi: 10.1038/s43588-021-00073-4, PMID: 38217213

[ref69] Welinder-Olsson C. Kjellin E. Badenfors M. Kaijser B. (2000). Improved microbiological techniques using the polymerase chain reaction and pulsed-field gel electrophoresis for diagnosis and follow-up of Enterohaemorrhagic *Escherichia coli* infection. Eur. J. Clin. Microbiol. Infect. Dis. 19, 843–851. doi: 10.1007/s100960000380, PMID: 11152309

[ref70] Wick R. R. Holt K. E. (2022). Polypolish: short-read polishing of long-read bacterial genome assemblies. PLoS Comput. Biol. 18:e1009802. doi: 10.1371/journal.pcbi.1009802, PMID: 35073327 PMC8812927

[ref71] Wick R. R. Judd L. M. Cerdeira L. T. Hawkey J. Meric G. Vezina B.. (2021). Trycycler: Consensus long-read assemblies for bacterial genomes. [Epubh ahead of preprint]. doi: 10.1101/2021.07.04.451066 PMC8442456 34521459

[ref72] Wick R. R. Judd L. M. Holt K. E. (2023). Assembling the perfect bacterial genome using Oxford Nanopore and Illumina sequencing. PLoS Comput. Biol. 19:e1010905. doi: 10.1371/journal.pcbi.1010905, PMID: 36862631 PMC9980784

[ref73] Yoon S. H. Ha S. M. Kwon S. Lim J. Kim Y. Seo H.. (2017). Introducing EzBioCloud: a taxonomically united database of 16S rrna gene sequences and whole-genome assemblies. Int. J. Syst. Evol. Microbiol. 67, 1613–1617. doi: 10.1099/ijsem.0.001755, PMID: 28005526 PMC5563544

[ref74] Zamora L. Fernández-Garayzábal J. F. Svensson-Stadler L. A. Palacios M. A. Domínguez L. Moore E. R.. (2012). *Flavobacterium oncorhynchi* sp. nov., a new species isolated from rainbow trout (*Oncorhynchus mykiss*). Syst. Appl. Microbiol. 35, 86–91. doi: 10.1016/j.syapm.2011.11.007, PMID: 22227311

[ref75] Zhao W.-H. Hu Z.-Q. (2010). β-Lactamases identified in clinical isolates of *Pseudomonas aeruginosa*. Crit. Rev. Microbiol. 36, 245–258. doi: 10.3109/1040841X.2010.481763, PMID: 20482453

[ref76] Zimin A. V. Marçais G. Puiu D. Roberts M. Salzberg S. L. Yorke J. A. (2013). The MaSurca genome assembler. Bioinformatics 29, 2669–2677. doi: 10.1093/bioinformatics/btt476, PMID: 23990416 PMC3799473

[ref77] Zimin A. V. Salzberg S. L. (2020). The genome polishing tool Polca makes fast and accurate corrections in genome assemblies. PLoS Comput. Biol. 16:e1007981. doi: 10.1371/journal.pcbi.1007981, PMID: 32589667 PMC7347232

