# Supplementary figures and images for: Description of Pseudomonas imrae sp. nov., carrying a novel class C β-lactamase gene variant, isolated from gut samples of Atlantic mackerel (Scomber scombrus)
Source: Front Microbiol. 2025 Apr 14;16:1530878. doi: 10.3389/fmicb.2025.1530878 (PMC12057487; doi:10.3389/fmicb.2025.1530878)

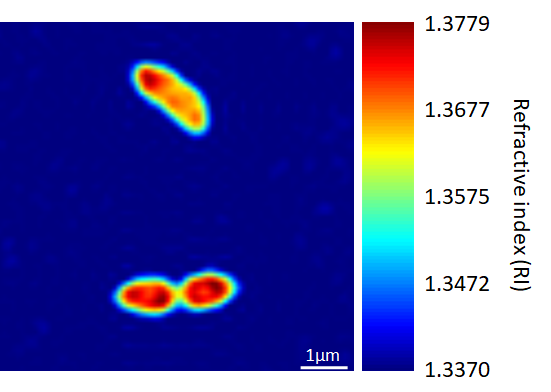

Supplement: SUPPLEMENTARY FIGURE 1 — Reconstruction of Pseudomonas imrae strain 16FHM2T using its refractive index, obtained with holotomographic imaging. [file Image_1.tif]
